# Epidemiology and cost of seasonal influenza in Germany - a claims data analysis

**DOI:** 10.1186/s12889-019-7458-x

**Published:** 2019-08-13

**Authors:** Stefan Scholz, Oliver Damm, Udo Schneider, Bernhard Ultsch, Ole Wichmann, Wolfgang Greiner

**Affiliations:** 1 School of Public Health, Bielefeld University, Universitätsstr. 25, 33615 Bielefeld, Germany; 2 Centre for Health Economic Research Hanover (CHERH), Leibniz University Hanover, Hanover, Germany; 3 Techniker Krankenkasse (TK), Hamburg, Germany; 4 Immunization Unit, Robert Koch Institute (RKI), Berlin, Germany

**Keywords:** Seasonal influenza, Epidemiology, Health economics, Cost of illness, Claims data

## Abstract

**Background:**

Seasonal influenza contributes substantially to the burden of communicable diseases in Europe, especially among paediatric populations and the elderly. The aim of the present study was to estimate the incidence of seasonal influenza in Germany, the probabilities of related complications and the economic burden of influenza per case and on a population level for different age groups.

**Methods:**

Claims data from 2012 to 2014 from > 8 million insured of a large German sick-ness fund were analysed. A matched case control study was used on a sub-sample of 100,000 influenza cases to calculate complication rates for ear infections/acute otitis media (AOM) and community-acquired pneumonia (CAP) as well as resource use and costs for seven age groups.

**Results:**

Incidence of seasonal influenza varies between the years and is highest among infants and children 2 to 5 years of age. AOM is more likely in the younger age groups with up to 14% more patients in the influenza group than in the control group. CAP is more frequently observed in the younger age groups and in influenza patients 60 years and older. The manifestation of one influenza complication (AOM or CAP) significantly in-creases the occurrence of a second complication (AOM or CAP). The economic burden per case is highest in infants (€251.91) and persons over 60 years of age (€131.59).

**Conclusion:**

The burden of influenza is highest among infants and young children, which is also reflected in the economic burden. Influenza related costs per case are nearly double for infants compared to persons over 60 years of age.

**Electronic supplementary material:**

The online version of this article (10.1186/s12889-019-7458-x) contains supplementary material, which is available to authorized users.

## Background

Seasonal influenza is an acute viral respiratory infection that poses a substantial burden on individuals, populations, and health care systems every year. It can cause mild to severe illness and can present with symptoms such as fever, muscle aches, fatigue, headache, and respiratory symptoms. While most people recover from influenza illness within a few days [1], some may develop severe complications including primary influenza virus pneumonia or secondary bacterial pneumonia [2]. Infected children often suffer from AOM [3, 4]. Severe non-pulmonary complications include cardiovascular and neurologic complications, which are, however, widely under-recognized since they are often not clearly linked to a previous influenza infection [5]. People at high risk for complications include very young children, older adults, pregnant women, nursing home residents, and people with chronic medical conditions or a compromised immune system [6].

Several vaccines are available to prevent influenza virus infections, and in many industrialized countries, high risk groups are targeted by national immunization programs or immunization recommendations [7]. In Germany, influenza vaccination is recommended for all persons above 60 years of age, pregnant women, high-risk persons (e.g. diabetes patients) and health care professionals [8]. The recommendation did not specify whether a tri- or tetra-valent vaccine should be used until 2018. Since then, a tetra-valent vaccine is recommended. The protection through the vaccination is also dependent on the vaccination rates. A decrease of the vaccination rates can be observed for Germany [9]: In the 2008/09 season 47.9% of persons above 60 years of age were vaccinated while only 34.8% were vaccinated in the 2016/17 season.

In this context, estimating the influenza-related disease burden in terms of morbidity, mortality, and economic consequences is an important contribution to the decision-making process regarding policies around influenza immunization [10]. In many countries, data on disease burden caused by seasonal influenza viruses are mainly gathered through national surveillance systems. Such systems often have a strong focus on assessing the role of influenza in acute respiratory illnesses (ARI) or influenza-like-illnesses (ILI) or report the proportion of cases with laboratory-confirmed influenza (LCI). In addition, using the example of the United States, combining routinely collected surveillance data with results of outbreak investigations and health care surveys allows for estimating symptomatic community illnesses, outpatient medical visits, hospitalizations, and excess deaths related to seasonal influenza [11]. The estimation of some of these outcomes, particularly influenza-associated hospitalization rates and excess deaths, commonly involves the use of statistical models [12]. The focus of the German surveillance system also includes estimated excess rates for influenza-associated outpatient medical visits, hospitalizations, cases of absenteeism, and deaths [13]. However, these reports do not include estimates for complications such as AOM or CAP and provide therefore rather partial insights in the burden of seasonal influenza. Furthermore, the German surveillance reports provide no information on the related direct and indirect cost of influenza. To close this evidence gap the aim of this study is to estimate the epidemiology of influenza and associated costs using claims data of a German sickness fund.

## Methods

### Data

Claims data from a large German statutory sickness fund (2012: 8,038,608 insured, 2013: 8,412,199, 2014: 8,849,736) covering a 3-year period (January 2012 to December 2014) was used to conduct the analysis. During this period, influenza strains were evenly distributed among the samples taken for the virological surveillance of the National Reference Center for Influenza for the 2012/13 season (A (H3N2): 31%; A (H1N1): 34; B-lineage: 35%). In the two following seasons, A (H3N2) was responsible for most cases (61 and 62%, respectively) with A (H1N1) being found in 30 and 15% and the two strains from the B-lineage in 9 and 23% of the cases. Yamagata has been the dominant B-strain in all seasons and has also been included in the trivalent vaccine [14]. The claims data set covers a broad range of medical and economic information from insured such as diagnoses, resource use and cost data, but primarily serves sickness funds for accounting purposes. Statutory sickness funds are covering 90% of the German population and cover the expenditure for health services from inpatient and outpatient sector as well as pharmaceuticals, remedies and aids and other services.

Due to the nature of the dataset, no laboratory-confirmed definition of influenza cases could be used. Instead, an influenza or ILI case was defined via ICD-10 codes J09 to J11 which were either documented in the dataset as a main inpatient diagnosis or a “secured” or “suspected” outpatient diagnosis. Influenza incidence estimates were based on all insured, while complication rates, costs, and work days lost were analysed via a matched case-control design. A sample of 100,000 persons being representative by age group, sex and insurance status for all insured diagnosed with influenza were drawn from the total population of insured, and controls without influenza diagnosis were matched one-to-one with replacement by 27 age groups, sex, category of insured persons (e.g. unemployed, employed, student), and outpatient pharmaceutical costs of the previous year with a caliper of ±10% as a proxy for comorbidity. Potential complications of influenza were identified using the ICD codes H65, H66, and H67 for ear infections or AOM and J10.0, J11.0, and J12 to J18 for CAP.

### Analysis

The calculation of the incidence was based on all insured of the sickness fund. All other analyses were based on the data of those insured for which a match could be identified. As outpatient diagnosis information in German claims data are usually documented on a quarterly base, the exact day of the influenza diagnosis cannot be determined. Therefore, the quarter of the initial influenza diagnosis (index quarter) and the following quarter were used to compare the documented diagnoses, resource use, and costs of the influenza patients and their matched controls. This means that the resource use and costs are calculated for half a year. To minimize the influence of outliers and to ensure meaningful results for rare events (e.g. influenza-related hospitalizations), the initial 27 age groups that were used for matching purposes were merged into six groups (“0 to 1”, “2 to 5”, “6 to 9”, “10 to 17”, “18 to 34”, “35 to 59”, “60 plus”) for the actual analysis. The choice of the age groups was based on to the different levels of the German educational system.

For the calculation of complication rates, the number of persons with at least one diagnosis of AOM or CAP during the index quarter and the following quarter was counted. Age and year-specific complications rates were calculated for both the influenza group and the control group. Influenza-attributable complication rates were then derived from the difference between the groups.

The calculation of influenza-attributable resource use and costs differed by health care sector. For inpatient treatment and sick leave, the corresponding principal diagnosis was identified directly from the claims data, i.e. ICD codes were available for each item of resource use on a daily base. Hence, there was no need of using the control group data for inpatient costs and sick leave. Inpatient costs could directly be determined by the payments of the sickness fund to the hospitals. For the indirect costs due to sick leave, the number of days absent from work was retrieved from the claims data. As income was not included in the dataset, the age-specific, average daily income of an employee (including part-time employees) [15] was used and multiplied with the number of days of the sick leave. Indirect costs were not calculated for the three youngest age groups and only for employed persons in the other age groups.

For outpatient physician consultations and prescribed pharmaceuticals, a direct link between diagnosis information and resource use or cost data was not possible. Therefore, respective influenza-attributable resource use had to be calculated via the matched case-control approach, i.e. excess resource use and costs were estimated by calculating the difference between the influenza group and the control group for the index quarter and the following quarter. The analyses of pharmaceuticals were further restricted to a list of relevant ATC codes for the treatment of influenza and related complications, which was adopted from Ehlken et al. [16]. The relevant ATC-codes are listed in Table 1.

**Table 1:** ATC-codes related to the treatment of influenza and included in the analyses

Substance	Code
Analgesics and antipyretics	N02B
Anti-inflammatory and anti-rheumatic products, non-steroids	M01A
Nasal decongestants	R01AA, R01AB
Throat preparations	R02A
Cough and cold preparations	R05
Antibiotics	J01A, J01C, J01D, J01F, J01 M
Antivirals	J05AH

The analyses were stratified by age groups to reflect the different risk-groups and by year to account for varying influenza activity in different seasons. Detailed results for years and age groups can also be found in a table in the Additional file 1.

### Statistics

Cost data from 2012 and 2013 were adjusted to the price level of the reference year 2014. The health-specific consumer price index of the German federal statistical office was used to adjust prices between 2012 and 2013 (− 3.7%) and between 2013 and 2014 (2.0%). Differences between the influenza group and the control group were tested using the two-sample, two-sided t-test for samples with unequal variances. Tests across k > 2 groups (e.g. comparison across years) were corrected for multiple testing by adjusting the alpha level by alpha/k according to the Bonferroni correction [17]. Analyses were performed with R (Version 3.4.1) and Microsoft Excel (Version 2013). We followed the German guidelines on routine data analysis [18].

## Results

### Study population

Across all 3 years, a match was found for 95,089 of the 100,000 insured sampled with influenza. The comparison of the sample characteristics between the matched and unmatched population can be found in Table 2. There is a significant difference between the two groups: Persons for which no matching partner could be found, are significantly younger (one-sided t-test), have a slightly different sex distribution, a different distribution of the category of insured persons, and show an unequal distribution over the years (chi-squared tests). The final dataset contained 190,178 persons with 95,089 influenza cases and 95,089 controls.

**Table 2:** Characteristics of the study population, stratified by successful matching

Variable	Level	Total sample	Cases (*N* = 95,089)	Controls (*N* = 95,089)	No matching partner found (*N* = 4911)	*p*-value for difference matched vs. unmatched
Year	2012 (%)	25,223 (25.2%)	23,702 (24.9%)	23,702 (24.9%)	1521 (31.0%)	< 0.001
	2013	50,799 (50.8%)	48,786 (51.3%)	48,786 (51.3%)	2013 (41.0%)	
	2014	23,978 (24.0%)	22,601 (23.8%)	22,601 (23.8%)	1377 (28.0%)	
Age in years	Mean (SD)	34.01 ()	34.84 (19.58)	34.84 (19.58)	19.74 (20.53)	< 0.001
Age group	0 to 1 (%)	1935 (1.9%)	1693 (1.8%)	1693 (1.8%)	242 (4.9%)	< 0.001
	2 to 5	7755 (7.8%)	6324 (6.7%)	6324 (6.7%)	1431 (29.1%)	
	6 to 9	5415 (5.4%)	4860 (5.1%)	4860 (5.1%)	555 (11.3%)	
	10 to 17	9184 (9.2%)	8405 (8.8%)	8405 (8.8%)	779 (15.9%)	
	18 to 34	25,917 (25.9%)	25,059 (26.4%)	25,059 (26.4%)	858 (17.5%)	
	35 to 59	40,261 (40.3%)	39,531 (41.6%)	39,531 (41.6%)	730 (14.9%)	
	60 plus	9533 (9.5%)	9217 (9.7%)	9217 (9.7%)	316 (6.4%)	
Sex	Male (%)	49,103 (49.1%)	46,574 (49.0%)	46,574 (49.0%)	2529 (51.5%)	0.001
	Female	50,897 (50.9%)	48,515 (51.0%)	48,515 (51.0%)	2382 (48.5%)	
Category of insured persons	Employed (%)	80,312 (80.3%)	78,390 (82.4%)	78,390 (82.4%)	1922 (39.1%)	< 0.001
	Self-employed	3109 (3.1%)	2581 (2.7%)	2581 (2.7%)	528 (10.8%)	
	Unemployed	4720 (4.7%)	3770 (4.0%)	3770 (4.0%)	950 (19.3%)	
	Social security beneficiary	75 (0.1%)	11 (0.0%)	11 (0.0%)	64 (1.3%)	
	Student	1453 (1.5%)	1239 (1.3%)	1239 (1.3%)	214 (4.4%)	
	Retired	7766 (7.8%)	7211 (7.6%)	7211 (7.6%)	555 (11.3%)	
	Other	2565 (2.6%)	1887 (2.0%)	1887 (2.0%)	678 (13.8%)	
Pharmaceutical costs previous year	Mean (SD)	311.74€ (2486.59€)	255.63€ (1238.33€)	256.14€ (1242.43€)	2077.62€ (12,240.23€)	< 0.001

### Epidemiology

#### Influenza incidence

**Fig. 1:**
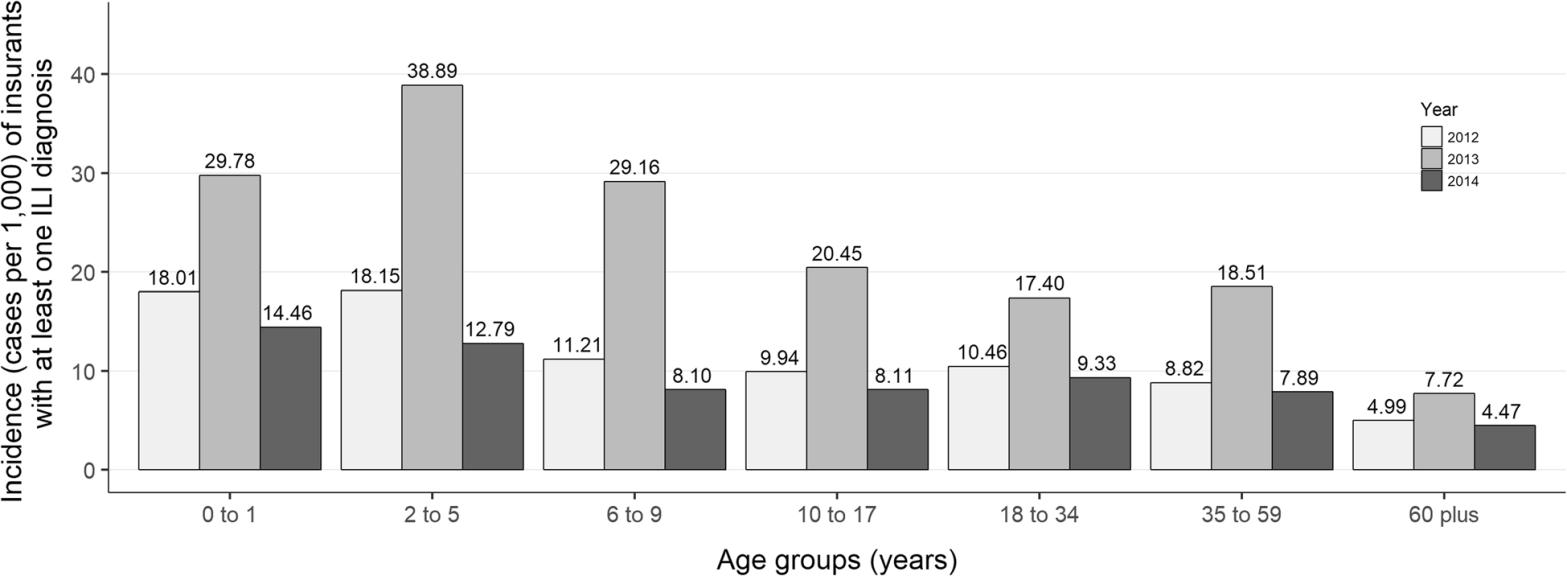
Incidence per 1000 insured persons for the years 2012 to 2014 for seven different age groups

The overall influenza incidence was 9.26, 17.90, and 8.00 cases per 1000 insured persons for the years 2012, 2013, and 2014, respectively. Across all 3 years, incidence was highest in children aged < 6 years and lowest in persons aged 60 years and above (see Fig. 1). The highest incidence was found for children aged 2 to 5 years in the year 2013 with 38.89/1000 insured children of the same age. The lowest incidence was found in persons aged 60 years and older in 2014.

#### Complications

**Fig. 2:**
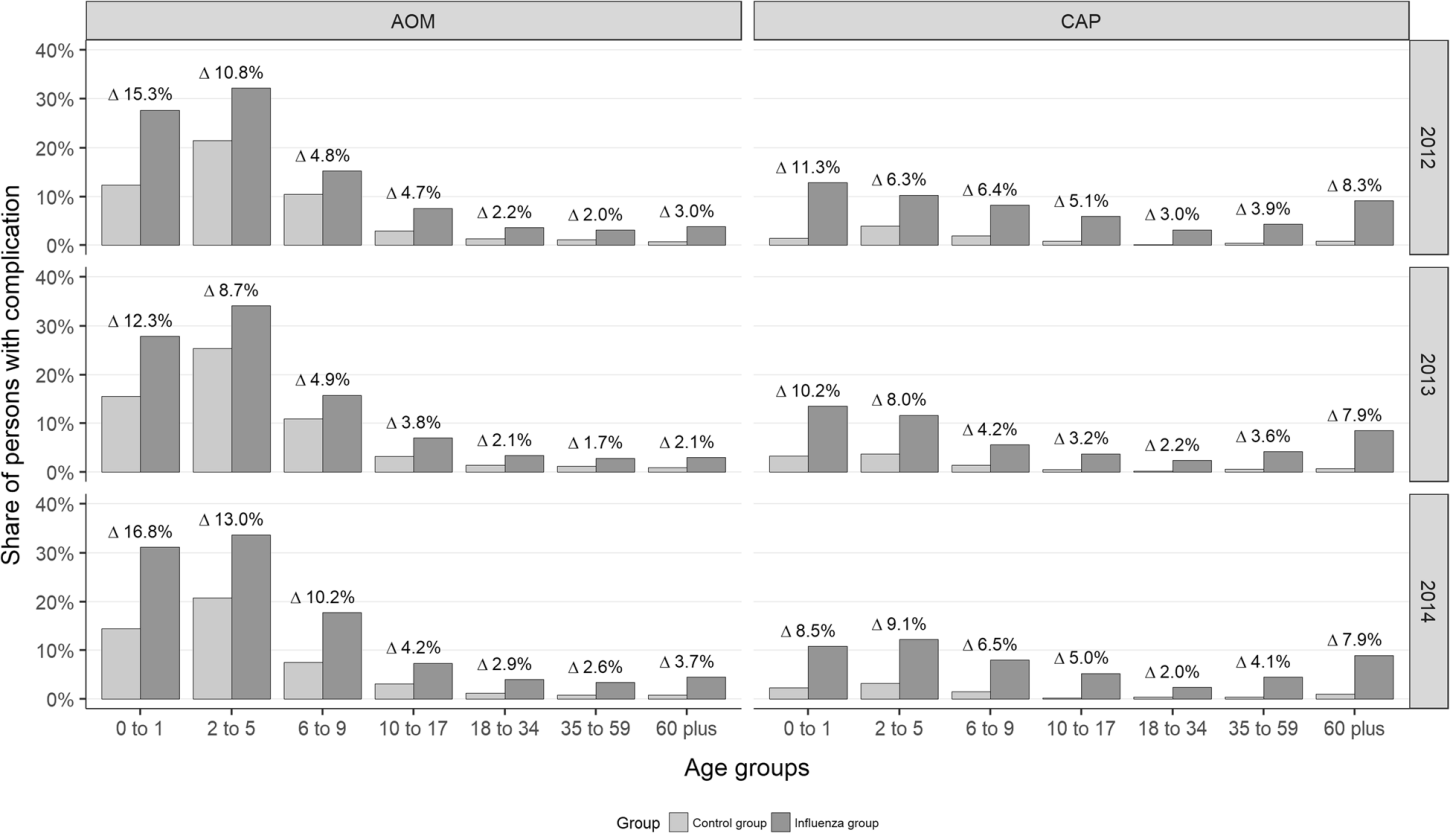
Complication rates for AOM and CAP in the influenza group and the control group for the years 2012 to 2014

To estimate the influenza-attributable complication rates, the incidence of AOM and CAP was assessed for the influenza and the control group separately (Fig. 2). Generally, the incidence of AOM and CAP was higher in the influenza as compared to the control group for each year and age group. On average, the influenza-attributable complication rate was 3.30% for AOM and 4.28% for CAP with no significant differences across the years. The highest influenza-attributable complication rate was found for AOM in children aged 2 to 5 years in 2014. Across the total study period, the complication rates for AOM attributable to influenza were 14.41, 10.07, 5.78, 4.12, 2.33, 1.99, 2.76, and 3.29% from the youngest to the oldest age group. The corresponding values for CAP were 10.04, 7.80, 5.08, 4.02, 2.35, 3.77, 8.01, and 4.28%.

Across all study years and age groups, the concordance of both complications was observed significantly more frequent than expected from their respective marginal probabilities (Chi-squared test). Multiplying the probability of AOM with the probability of CAP yields 328 cases with both complications in the influenza group and 27 cases in the control group, which were to be expected from the marginal probabilities of both complications. However, 704 cases with both complications were observed in the influenza and 142 in the control group. Because of the low cell counts, no tests were performed stratified by years and age groups.

#### Hospitalization

The dataset contained 458 persons (0.48%) who were hospitalized for influenza as the principal diagnosis across all years and age groups. Regarding the disease severity, the year 2013 does not only show a higher incidence but also a higher share of persons being hospitalized for influenza (2012: 0.39%; 2013: 0.58%; 2014: 0.37%). The age-specific risk of hospitalization was highest for infants (2.24%) and young children (2 to 5 years: 1.15%) and decreased over age to about 0.30% for persons aged 35 to 59 years. Persons aged 60 years and above showed an increased probability of hospitalization of 0.84% as compared to younger adults.

The small number of inpatient stays for a principal diagnosis of influenza (e.g. only six persons aged 10 to 17 years in 2014) counteracts the discovery of significant trends in the estimates of the length of stay (LOS). The average LOS due to influenza (principal diagnosis) was 4.47 days. The oldest age group showed the longest LOS with 7.05 days, followed by infants aged < 1 year with 4.58 days.

For influenza patients with complications, the risk of being hospitalized was dependent on the type of complication. In both groups (influenza and control), only 40 patients diagnosed with an AOM received inpatient treatment because of AOM, and no significant difference could be found between the groups (Z-test for proportions, alpha level 0.05). This corresponds to a hospitalization rate of 0.45% of persons with AOM in the influenza group and 0.33% of persons with AOM in the control group. Because of the low number of observations, no time or age-specific trend was identified (data shown in the Additional file 1). With 6.85% in the influenza group and 5.49% in the control group, hospitalization rates for persons with CAP were much higher as compared to AOM.

Similar to influenza, trends in the LOS data for patients with AOM were hardly recognizable because of the low number of hospitalizations. Overall, the 29 hospitalized patients with AOM in the influenza group received on average 4.10 days of inpatient care, compared to 3.82 days for the 11 patients in the control group. As more persons with CAP were hospitalized, an age-specific U-shaped trend could be seen for the control (*n* = 42) and the influenza group (*n* = 331). Especially infants with CAP in the influenza group showed a prolonged LOS of 5.62 days in comparison to the control group.

### Resource use and costs

#### Outpatient sector

**Fig. 3:**
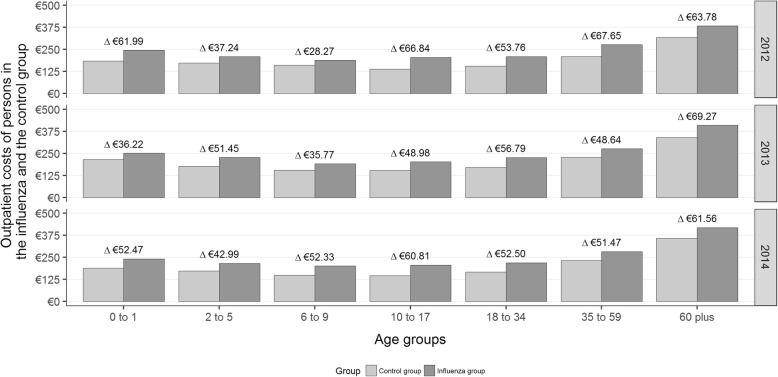
Outpatient costs in Euro (reference year 2014) of patients in the influenza (dark grey) and control (light grey) group by age group and year

Persons in the influenza group showed on average 4.60 outpatient physician contacts and caused average costs of €259.75 compared to 3.47 contacts and costs of €205.80 of the average person in the control group. As depicted in Fig. 3, there was a slight increase in outpatient costs with increasing age. Differences between the influenza and control group were significant at a 0.05-level (two-sided t-tests).

When selecting only influenza patients with complications and their matched controls, patients with an AOM diagnosis had 5.20 visits causing an average €297.13 in the influenza group and 3.37 visits causing €196.19 in the control group. Costs in the control group were only higher than for the influenza group in the year 2012 in the age group of “6 to 9 years”. The difference of €3 was not significant (*p*-value: 0.239).

#### Inpatient sector

The average inpatient costs per influenza hospitalization measured via the principal diagnosis were €2033 (SD €2952) with very little variation between the years (2012: €1948, 2013: €2079, 2014: €1974). In 2012 and 2013, age-specific costs followed a U-shape with higher costs in the youngest and oldest age groups and lower costs in the groups in the middle. This resembles the LOS trend found in those years. Costs were relatively stable across the age groups in 2014, which can be explained by a higher LOS in the middle age groups coinciding with lower prices for the coded diagnosis-related groups (DRGs) in those age groups. No trend for the average costs could be identified over the years.

The average inpatient costs for patients with AOM in the influenza group were €1632 (SD: €423) and were significantly different from the average costs of €2083 (SD: €532) in the control group (2-sided t-test; *p*-value 0.020). Again, the small number of AOM overall makes the interpretation of the stratified analyses difficult (detailed numbers can be found in the Additional file 1). Inpatient costs for CAP do not differ significantly between the influenza and the control group (€3995 vs. €2789, p-value 0.089). While the standard deviations were relatively small for AOM, the variation in costs of patients with a CAP complication in the influenza group were very high with a standard deviation of €12,002, leading to the difference of €1206 to be statistically insignificant. This is mainly explainable by the influenza-related hospitalization of a pre-term baby with multiple, congenital malformations with a LOS of 101 days in the influenza group in the age group “0 to 1 years” in the year 2013 which caused €157,463.40.

#### Prescription medication

Of all patients in the influenza group, 57.4% received influenza-related drugs, compared to 32.4% of in the control group. This corresponds to 0.65 more medications on average and €6.23 in the influenza group (€12.54 vs €6.31). Differences in the number of medications and costs were significant for all age groups across all years at a 0.05 alpha level, and there were only minor differences over time. While the percentage of persons with medication and the total pharmaceutical costs increased with age, no clear age-related trend could be identified for the differences between the influenza and control groups.

The number of medications for patients with AOM was significantly higher in the influenza group compared to the control group (3.39 vs. 1.42; *p*-value 0.000), which can also be seen in the cost difference of €15.46 between the groups. For CAP, the difference in prescriptions was slightly lower (1.53; p-value: 0.000) compared to AOM, but the average CAP patient in the influenza group had higher costs of €17.58 (p-value: 0.000) compared to the average patient in the control group.

#### Sick leave and indirect costs

On average 33.44% of patients in the influenza group were on sick leave for influenza for 6.71 days. In this estimate, the denominator includes also non-working persons. Looking at the subset of members of the insurance (i.e., the person/s in a family paying the premium) whose insurance status is “employed”, the percentage of persons on sick leave for an influenza diagnosis increases to 54.9% with a mean duration of 6.87 days. For all insured, the number of working days lost increases with age from 5.63 days for “18 to 34” to 8.25 days for “60 plus”. The indirect costs associated with this sick leave are €576.54 per case on average. For each age group, the costs are dependent on the number of working days lost, the income and the percentage of persons in the labour force in each age group, resulting in €251.36 for the age group “10 to 17”, €370.80 for “18 to 34”, €673.84 for “35 to 59” and €569.46 for the age group “60 plus”.

#### Total direct cost

**Fig. 4:**
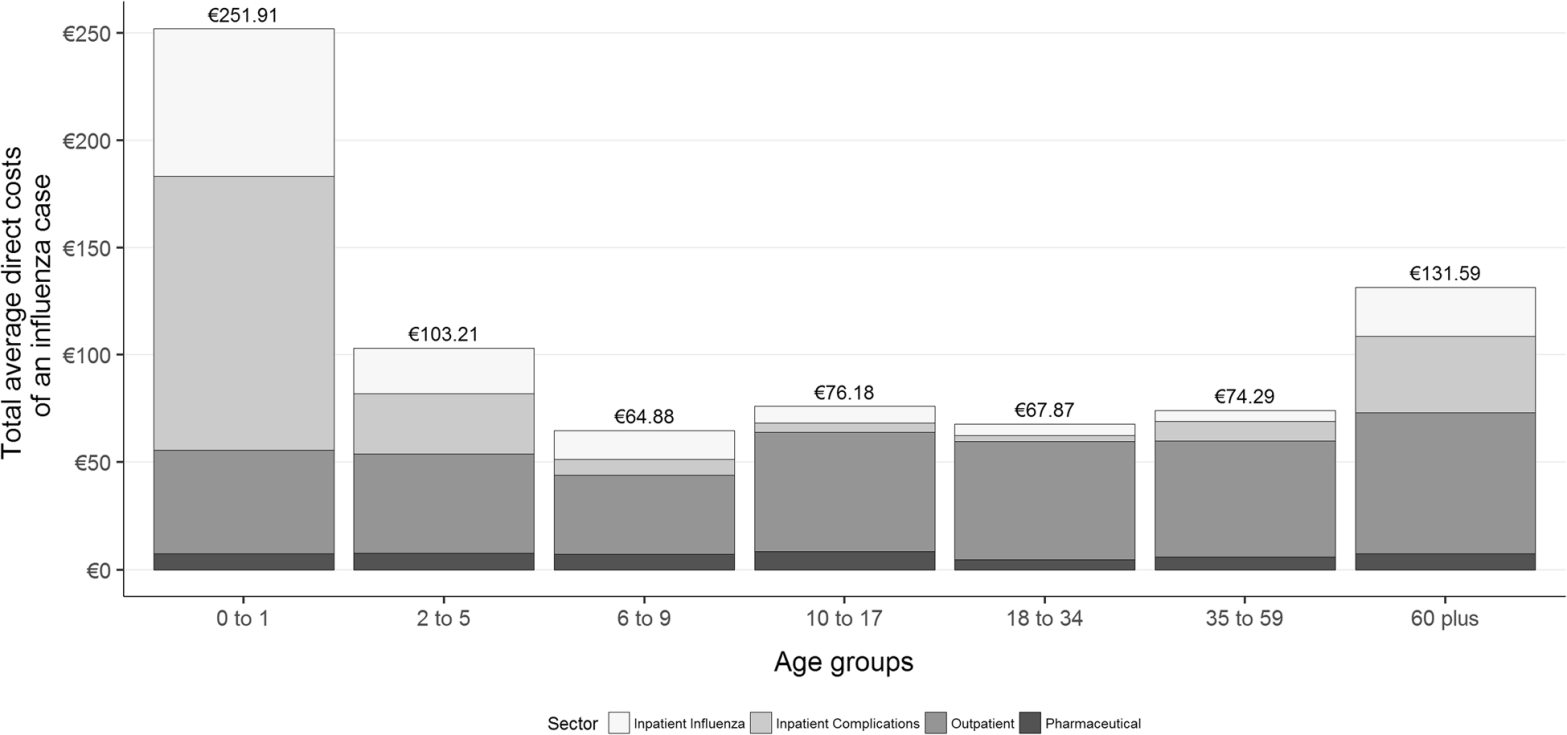
Total, average, influenza-attributable costs per influenza case

The total direct costs per average influenza case in our sample were €82.90, with higher costs in the youngest and oldest age groups. As can be seen from Fig. 4, the higher overall costs in those groups were mainly caused by the higher costs for inpatient care for complications and for influenza itself. The positive entries in the covariance matrix (see Additional file 1) between all cost categories suggest that higher costs in one sector are correlated with higher costs in other sectors. For example, the strongest, positive correlation between costs for pharmaceuticals and outpatient costs could be explained by more outpatient visits leading to more prescribed medications.

Assuming that the influenza incidence in this study population corresponds to the incidence in the total population of the sickness fund providing the data, this would result in 2,832,610 cases of influenza in the three-year period. Based on our calculations, in 2013 nearly twice as many cases have occurred when compared to 2012 and 2014, respectively (1,443,238 vs. 745,845 and 649,334 cases). Multiplying the age-group specific costs with the expected number of influenza cases in the general German population yields total costs of €78,278,429 per year (ranging from €52,879,376 in 2014 to €120,537,590 in 2012).

## Discussion

Our results confirm well-established differences in the incidence of seasonal influenza by season and by age groups. Furthermore, our analyses show that complication rates for influenza-attributable AOM and CAP are highly age-dependent. We found no significant differences of the complication rates between the three seasons included in this analysis. We were also able to show that the occurrence of both complications in the same patient is significantly more frequent than it could be expected from the marginal probabilities of the complications. The youngest and oldest age groups carried the highest burden of influenza measured by incidence and complication rates. Regarding the economic burden, the presented results suggest no strong cost differences per case between the years. Analogously to the disease burden, the youngest and oldest age group cause the highest mean costs among all age groups. Costs for outpatient care and pharmaceuticals are relatively similar for all age groups. Cost differences result from higher costs of inpatient care for influenza itself and influenza-related complications, respectively.

With regard to the incidence estimates, our results are comparable to the findings of the German Influenza surveillance at the Robert Koch-Institute. The four reports for the seasons 2011/12, 2012/13, 2013/14 and 2014/15 [19–22] estimate the number of influenza-related excess consultations per 100,000 inhabitants also to be highest among the youngest age-group and to be the lowest in the age-group of people above 60 years of age. Our findings are also in line with the severity of the different seasons, i.e. the reports show the highest number of excess consultation in the year 2013 and a fairly equal number in the years 2012 and 2014.

The results presented on resource use and costs in this study can only to some extent be compared to previous studies for Germany. The only two recent studies on influenza-associated disease burden and costs that were identified for Germany differed in their scope and the applied methods [16, 23]. While Ehlken et al. did not calculate incidence rates, our incidence estimates of 0.93% in 2012 and 1.73% in 2013 are comparable to the estimate of 1.7% estimated by Haas for the 2012/13 season. The complication rates for AOM and CAP calculated in our study show a similar trend across the age groups as compared to the figures presented by Haas et al. but each with a higher level. For example, Haas et al. estimated the influenza-attributable CAP to be 2.3% compared to 4.28% in our study. Ehlken et al. reported a complication rate of 1.6% for CAP in children (5.86% in our study) and 5.4% in adults (3.82%). The difference between the studies may be explained by relatively low numbers of CAP cases in all three studies. For AOM, Ehlken et al. found higher rates for children (13.4% vs. 7.09% in our study) but lower rates for adults (0.9% vs 2.20%). The complication rates for AOM as found by Haas et al. correspond well across the age groups, although on a lower level. For instance, for the age group “0 to 1”, the authors found a difference of 11.0% between influenza and control group compared to 14.41% in our study. This might be caused by different definition of the complications with regard to the selected diagnoses in the inpatient and outpatient sector documented in German routine data. Differences to Ehlken et al. might also be caused by differences in season-specific influenza epidemiology, e.g. the dominant strain circulating or the match of the seasonal vaccine, as the studies cover different seasons.

Regarding the cost estimates, the study by Ehlken et al. estimated the cost of one influenza episode to be €514 for adults and €105 for children from a societal perspective and €59 and €66 from a third-party payer perspective. Unfortunately, Haas et al. did not provide total cost estimates per influenza case or episode, but their estimates on the costs for outpatient care exceed the estimates of Ehlken et al. and our study fourfold (€224 vs. €34.51 vs. €53.95, respectively). Also, the estimates of Haas et al. for the costs per inpatient influenza case exceed our estimates (€5832 vs. €2033). For the outpatient sector, this may be due to the missing control group in the study by Haas et al. The corresponding costs of just the influenza group in our study are €259.75. The difference in outpatient costs might be explained by outliers.

Our study has several limitations. Firstly, the time period covered by the data did not coincide with the start and the end of the influenza seasons. Hence, the year 2012 contains the later part of the 2011/12 season and the first part of the 2012/13 season. By the nature of claims data, our dataset only contains cases of influenza seeking medical attendance. Thus, we might underestimate the burden of the disease as symptomatic cases who do not seek medical attention are not considered in the analysis. More generally, the estimation of the burden of disease of influenza is challenging to assess, since definition and diagnostic procedures differ, information on seek of medical attendance partly lacks, and virus circulation and vaccine fit differ almost every year.

Further, no out-of-pocket payments that are not reimbursed by the German sickness funds are part of our analysis. In this context, it is important to mention that the definition of the influenza group – including only patients with a J09, J10 or J11 diagnosis – is rather conservative. In this context, the high percentage of controls with influenza-related medication might be an indicator although many of the pharmaceuticals listed in Table 1 are used for various other medical conditions, e.g. ibuprofen or other analgesics. Additionally, it is not possible to exclude biases that may arise from the population of the sickness fund which was used for the analysis in this study. However, the sickness fund covers nearly 9.98, 10.42 and 10.90% of the general German population in the years 2012, 2013 and 2014, respectively. Finally, we did not include cardiovascular or neurological complications for which the link to influenza is not firmly established. To detect those complications and estimate their economic burden, a larger dataset would be necessary. Hence, our estimates can be seen as conservative.

## Conclusions

In summary, our study suggests that the economic burden of influenza on a population level corresponds to the high non-economic burden of disease of influenza found for Europe [24]. Even if only direct costs are considered, our estimates of €78,278,429 per year are nearly as high as the estimates for herpes zoster and postherpetic neuralgia with €105 million [25] and amount to one third of the yearly spending on prostate cancer of €244 million in Germany [26]. Costs are especially high for persons being defined as “high risk” from a clinical perspective and among children 2 to 5 years old. Further research could focus on the cost-effectiveness of a general, seasonal vaccination in dependency of the age at administration of the vaccine.

## Additional file

Additional file 1: The supplement contains detailed results for each age group and year for incidence and costs. (XLSX 330 kb)

## Data Availability

The data that support the findings of this study are available from the Techniker Krankenkasse but restrictions apply to the availability of these data, which were used under license for the current study, and so are not publicly available. Data are however available from the authors upon reasonable request and with permission of the Techniker Krankenkasse.

## Abbreviations

AOM: Acute otitis media
ARI: Acute respiratory illnesses
ATC: Anatomical Therapeutic Chemical
CAP: Community-acquired pneumonia
DRG: Diagnosis related group
ICD-10: International Classification of Diseases, Version 10
ILI: Influenza-like-illnesses
LCI: Laboratory-confirmed influenza
LOS: Length of stay
SD: Standard deviation
